# Synergy Analysis Reveals Association between Insulin Signaling and Desmoplakin Expression in Palmitate Treated HepG2 Cells

**DOI:** 10.1371/journal.pone.0028138

**Published:** 2011-11-23

**Authors:** Xuewei Wang, Aritro Nath, Xuerui Yang, Amanda Portis, S. Patrick Walton, Christina Chan

**Affiliations:** 1 Department of Chemical Engineering and Materials Science, Michigan State University, East Lansing, Michigan, United States of America; 2 Genetics Program, Michigan State University, East Lansing, Michigan, United States of America; 3 Department of Biochemistry and Molecular Biology, Michigan State University, East Lansing, Michigan, United States of America; 4 Department of Computer Science and Engineering, Michigan State University, East Lansing, Michigan, United States of America; Center for Genomic Regulation, Spain

## Abstract

The regulation of complex cellular activities in palmitate treated HepG2 cells, and the ensuing cytotoxic phenotype, involves cooperative interactions between genes. While previous approaches have largely focused on identifying individual target genes, elucidating interacting genes has thus far remained elusive. We applied the concept of information synergy to reconstruct a “gene-cooperativity” network for palmititate-induced cytotoxicity in liver cells. Our approach integrated gene expression data with metabolic profiles to select a subset of genes for network reconstruction. Subsequent analysis of the network revealed insulin signaling as the most significantly enriched pathway, and desmoplakin (DSP) as its top neighbor. We determined that palmitate significantly reduces DSP expression, and treatment with insulin restores the lost expression of DSP. Insulin resistance is a common pathological feature of fatty liver and related ailments, whereas loss of DSP has been noted in liver carcinoma. Reduced DSP expression can lead to loss of cell-cell adhesion via desmosomes, and disrupt the keratin intermediate filament network. Our findings suggest that DSP expression may be perturbed by palmitate and, along with insulin resistance, may play a role in palmitate induced cytotoxicity, and serve as potential targets for further studies on non-alcoholic fatty liver disease (NAFLD).

## Introduction

Accumulation of lipids (primarily triglycerides) in hepatocytes is considered a hallmark and pre-requisite for development of non-alcoholic fatty liver disease (NAFLD) [1]. With increasing prevalence of obesity among adults and children, NAFLD has become the most common form of chronic liver disease in developed countries [2]. Studies have shown that diets rich in saturated fats contribute significantly towards increasing the risk of NAFLD [3], [4]. Consequently, a large number of studies have focused on the effects of free fatty acids (FFA) on hepatocytes to understand the pathogenesis of NAFLD and related liver diseases (reviewed in [5]). In recent years, systems biology approaches utilizing high-throughput (microarray) measurements have been applied to gain significant insights into the cytotoxic effects of FFAs on hepatocytes using either static [6], [7], [8], [9] or dynamic gene expression profiles [10]. While these efforts have focused predominantly on identifying individual target genes, some researchers have suggested a more complex scenario, whereby genes cooperate in regulating cellular events in response to FFA treatment [11].

In this study, we analyzed the cytotoxic effects of palmitate treatment, the most common FFA in the diet, on HepG2 cells. First, we selected a subset of genes affected by FFA treatment by mapping their gene expression to metabolite profiles [8], [12]. This allowed the integration of multi-level data and further helped to alleviate the computational burden associated with the analysis of a large set of gene expression data. Next, we used an integrative methodology to reconstruct a gene cooperation network using the concept of “Information Synergy” [13]. The underlying principle of information synergy states that if two genes cooperate to affect a phenotype, then the joint expression of these two genes should provide more information on the phenotype than the sum of the information contributed independently by each of the genes. Thus, the gain in information or information synergy could be used to quantitatively asses the cooperative effect of any two genes on a phenotype. To help elucidate the processes that may be altered we analyzed the pathways that were enriched in the network.

Our search for over-represented pathways in the synergy network recovered insulin signaling pathway as the most significantly enriched pathway. This is notable given the fact that almost all patients diagnosed with NAFLD have concomitant insulin resistance [14]. Ruddock *et al.* confirmed that palmitate treatment induces resistance to insulin signaling in hepatocytes [15]. We further expanded our search to the neighbor genes of the insulin signaling pathway in the synergy network and recovered desmoplakin (DSP), a junction protein, as the top neighbor. The DSP protein is an obligate component of functional desmosomes at intercellular junctions. DSP together with plakoglobin and plakophilin forms the intracellular desmosomal plaque [16], which serves as an anchor for keratin intermediate filament attachment [17]. In general, junction proteins are known to be inhibited during chronic liver diseases, including cirrhosis and hepatitis [18]. It has also been shown that the loss of expression and abnormal localization of junction proteins, including DSP, is correlated with progression of HCC [19]. However, the effects of palmitate on DSP protein expression have not been previously reported.

Here, we investigated the effects of palmitate treatment on the expression of DSP in HepG2 cells. Furthermore, since DSP was recovered as a neighbor of insulin signaling pathway, we analyzed the effect of insulin treatment on DSP expression. We found that palmitate treatment leads to the loss of DSP expression and treatment with insulin enhanced the recovery of DSP expression following palmitate treatment. Thus, our study indicates that the junction protein DSP, in synergy with insulin signaling, may be a novel target in the pathology of fatty liver disease.

## Materials and Methods

### Datasets

HepG2 cells purchased from American Type Culture Collection (ATCC) were exposed to different FFA treatments for 48 hours. Gene expressions of these treated cells were then profiled with cDNA Microarrays at the Van Andel Institute (Grand Rapids, MI) and pre-processed by GenePix Pro 5.0 (Microarray Acquisition and Analysis Software from Molecular Devices, LLC). The fluxes of 28 metabolites were measured using either HPLC or kits, and cytotoxicity was evaluated based upon LDH levels in the supernatants and in the cell lysates. The procedures of fatty acid treatments, microarray analysis, cytotoxicity and metabolic fluxes measurement were described in our previous works [8], [12]. The microarray dataset has been deposited to GEO website [20], with the query number of GSE26885.

### Gene selection based on the trend of the metabolites profiles

FFAs modulate multiple intracellular metabolic pathways, many of which are involved in the pathophysiology of fatty liver disease. The fluxes of various metabolites were used to identify four representative trends across the treatment conditions: bovine serum albumin (BSA, control), Palmitate and Oleate, as discussed in [21] (see Figure S1). The gene expressions were first logrithmized with base 2, and the expressions for the probes designed for same genes were averaged out. The genes annotated with unknown functions (i.e. “EST/hypothetical proteins” or “ORF of unknown functions”) were removed from the dataset. Then, the expression patterns of the remaining genes were matched to the four representative metabolite trends across BSA, Palmitate and Oleate. We further removed the genes not differentially expressed between any two of the three conditions based on two-sample t-test (p-value cutoff set at 0.05). Finally, 610 genes remained (see Table S1) for further analysis.

### Calculation of information synergy

An information-theoretic measure was used to quantify the synergy between the genes. Given two genes, G1 and G2, and a phenotype P (toxic or non-toxic in our case), the information synergy between G1 and G2 with respect to the phenotype P is defined as:
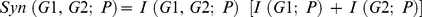
where I(G1;P) is the mutual information between G1 and P, I(G2;P) is the mutual information between G2 and P, and I(G1,G2;P) is the mutual information between (G1,G2) and P. This equation reflects the definition of synergy, the additional contribution provided by the “whole” as compared to the sum of the contributions of the individual “parts”. Mutual information (I) was calculated based on a clustering-based method for continuous expression data [22], on an information synergy scale of [−1 1]. A positive synergy score indicated that two genes jointly provided additional information on the phenotype, a negative synergy score indicated that the two genes provided redundant information about the phenotype, and a zero score indicated that the two genes provided no additional information on the phenotype.

A permutation test was performed to assess the statistical significance of the synergy score of the gene pairs. The phenotypes were randomly permutated to un-correlate the phenotype with the gene expression profiles, and then the synergy scores were re-calculated based on the permutated phenotype for all gene pairs. This process was repeated 1000 times to calculate the p-values of the synergy score for each gene pair. A Benjamin-Hochberg false discovery rate procedure [23] was applied to adjust the p-values for all the gene pairs and thereby control the expected false discoveries. Finally, 4376 out of the 185745 pairs from the 610 genes were identified as significant based on a cutoff of 0.05 for adjusted p-values.

### Statistical test for over-represented pathways and pathway-gene associations

A web platform GENECODIS [24] was used to identify the KEGG pathways overrepresented in the synergy network. The hyper-geometric test was used to calculate the p-values of the KEGG pathways in the network, followed by Benjiamin-Hochberg FDR control procedure to obtain adjusted p-values, and finally the adjusted p-value cutoff was set at 0.05 to determine the significantly over-represented pathways.

Likewise, hyper-geometric test was also used to determine the significant neighboring genes for each over-represented pathway in the network. Given one pathway and one of its neighboring genes in synergy network, the p-value of this pathway-gene association was calculated as below:
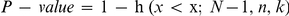



Where x is the number of pathway genes connected to the given gene in the network; *N* is the number of genes in the network; *n* is the number of pathway genes in the network; *k* is the number of genes connected to the given genes in the network. For each pathway, the p-values of its neighbor were adjusted by Benjiamin-Hochberg procedure, and the adjusted p-value cutoff was set at 0.05 to determine the significantly associated neighbors.

### Differential Correlation Network

A differential correlation network was constructed by obtaining gene-pairs which show significant difference in the correlation coefficients between phenotypes [25]. Pathway enrichment analysis and pathway-gene association analysis were performed as described above for synergy network.

### Cell culture and treatment

HepG2 cells were cultured in Dulbecco's Modified Eagle's Medium (DMEM) (Invitrogen) containing 10% fetal bovine serum (Invitrogen) and 2% Penicillin-streptomycin (Invitrogen). Cells were incubated at 37°C and in 10% CO_2_ atmosphere. Confluent cells were treated with 0.7 mM palmitate (Sigma) dissolved in DMEM culture medium for 48 hours. Cells were treated with 1 nM insulin in culture medium for 24, 48 and 72 hours. DMEM culture medium was used as control for all treatments.

### Immunofluorescence

Cells were fixed in 3.7% formaldehyde for 20 min. Cells were then washed twice with PBS and treated with 5% Triton-X for 10 min. at room temperature to permeabilize cell membrane. This was followed by washing twice with PBS and incubation with 1% BSA to block non-specific protein interactions. The cells were then incubated with 5 µg/ml anti-rabbit DSP primary antibody (Abcam ab71690) overnight at 4°C. After washing twice with PBS, cells were incubated in AlexaFluor-488 (Molecular Probes A11001) conjugated goat anti-rabbit secondary antibody (green) for 1 hour at room temperature. Cells were washed with PBS and cell nuclei were stained (blue) using DAPI (Molecular Probes D1306) at a concentration of 300 nM for 5 min. Coverslips were mounted in ProLong Gold (Molecular Probes P36934) and incubated in the dark for 24 hours.

Confocal imaging was performed on an Olympus FluoView 1000 Inverted IX81 microscope, using a 40X oil objective. Blue and green images were taken sequentially, using a Kalman average of 2X. The intensity graph was constructed by normalizing the total green fluorescence intensity to total blue fluorescence intensity (to account for the number of cells) in 3 separate fields of view for each sample. Multiple comparisons between fluorescence levels across different treatment conditions were performed using one-way ANOVA followed by Tukey's HSD post-hoc analysis with p-value cut-off set at 0.05.

## Results

### 1 Information synergy network

Based upon the concept of information synergy, we reconstructed a gene-cooperation network composed of 570 genes with 4376 connection edges. Topological analysis shows that this network follows a power-law distribution (see Figure S2) that is different from the bell-like distribution of a random network. The reconstructed network suggested potential gene targets and pathways that may play important roles in the induction of the cytotoxic phenotype.

### Positive information synergy indicates joint association with phenotype

**Figure 1:**
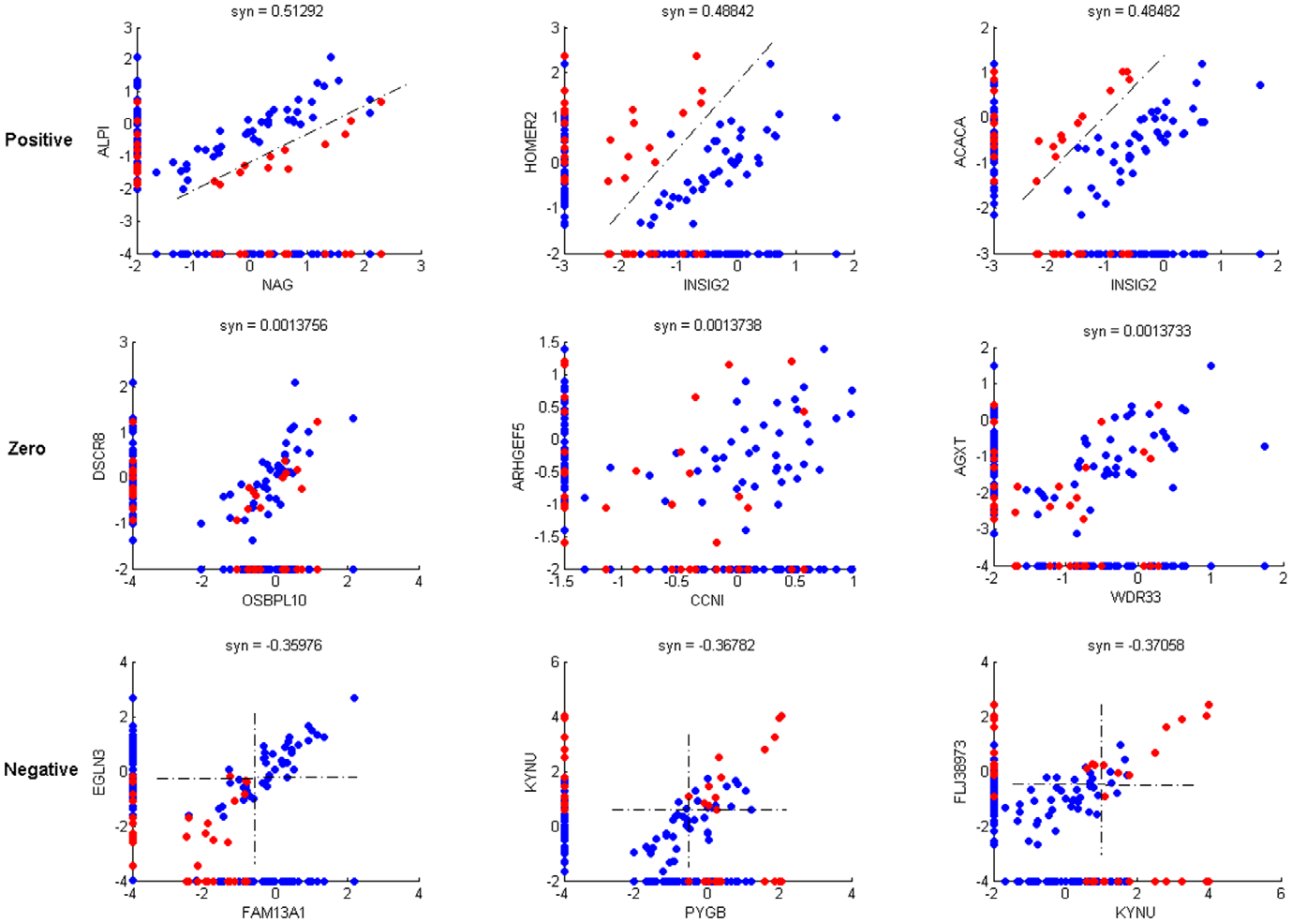
Joint expression of gene pairs with different information synergy. Blue and red points represent nontoxic and toxic samples, respectively. From top to bottom: Gene pairs with *positive* synergy jointly discriminate the phenotypes (indicated by the black dashed lines – joint information), whereas either of the genes alone provide little information in discriminating the phenotypes (indicated by the data points projected to the axes – marginal information). In contrast, genes with *zero* information synergy appear to be independent of the phenotypes, both jointly and marginally. Gene pairs with *negative* information synergy are marginally informative on the phenotype, such that either of the genes can provide information on the phenotype, but the additional information provided by the other gene does not enhance the discrimination of the phenotype, i.e. information provided by both genes about the phenotype is redundant.

We interrogated gene pairs in terms of their ability to jointly distinguish phenotypes. As described above, positive, zero and negative information synergies represent gene pairs that provide synergistic, no and redundant information on the phenotype, respectively. Examples of gene pairs for each level of information synergy, demonstrating how they jointly provide information on the phenotypes, are illustrated in Figure 1.

For the gene pairs with significant positive synergy in Figure 1 (top row), neither of the two individual genes was strongly correlated with the phenotype in the univariate marginal distribution. On the other hand, the gene *pairs* were significantly correlated to the phenotype, i.e. their expressions *jointly* help to distinguish the phenotypes. The two phenotypes are clearly discernible with the existence of a gap, previously denoted as a “gap” pattern [26]. Additional correlation patterns, including “substitute” and “on/off” [26], were also observed in a small number of positive gene pairs (see Figure S3 for explanations of these correlation patterns). Thus, both genes in the pair with positive synergy would have been ignored by a correlation based one-gene-at-a-time approach which considers only those genes that are highly correlated to the phenotypes.

### 2 Enriched pathways related to palmitate induced cytotoxicity

The pathways enriched in the synergy network were explored through enrichment analysis. 42 pathways were significantly enriched in the synergy network (see Table S2), and the top 10 pathways are listed in Table 1.

**Table 1:** Top ten KEGG pathways ranked by their p-values enriched in the synergy network.

Pathway names	#genes in network	p-value	Affected by Palmitate
04910: Insulin signaling pathway	15	6.68E-07	Known [15]
04520: Adherens junction	10	2.10E-05	Known [29], [30]
00310: Lysine degradation	7	0.00014	Known [12]
00330: Arginine and proline metabolism	6	0.00053	Known [12]
04916: Melanogenesis	8	0.00556	
04120: Ubiquitin mediated proteolysis	9	0.00644	Known [28], [65]
04010: MAPK signaling pathway	13	0.00658	Known [66]
05210: Colorectal cancer	7	0.00716	
04310: Wnt signaling pathway	9	0.00942	Known [67]
04810: Regulation of actin cytoskeleton	11	0.00981	Known [30], [68]

As seen in Table 1, cellular activities such as insulin signaling, adherence junction/cytoskeleton regulation, amino acid metabolism, and ubiquitin-mediated proteolysis are highly enriched in the synergy network. Indeed, it has been suggested in the literature that palmitate treatment affects several of these enriched pathways, including insulin signaling inhibited by palmitate in hepatomal cells [15], ER stress and the ubiquitin mediated protease pathways [27], [28], as well as adherence junction and cytoskeleton structure [29], [30]. Palmitate has been also shown to modulate the metabolism of various amino acids [12] which may be important players in palmitate-related cytotoxicity. For example, arginine metabolism provides substrate for nitric oxide synthetase (NOS) and palmitate treatment was found to enhance cell death by increasing NOS activity and NO production [31].

### 3 Pathway-gene association analysis

In this section, we investigated the associations between the enriched pathways and their neighbors in the synergy network. Neighbors were defined as genes in the network connected to at least one gene of the enriched pathway. The connections in the network represent cooperative relationships between the genes. Therefore, we assumed that the neighbor genes, especially those connected to multiple members of an enriched pathway may function cooperatively with the enriched pathway in the associated phenotype.

### Neighbor genes relevant to insulin signaling pathway

**Figure 2:**
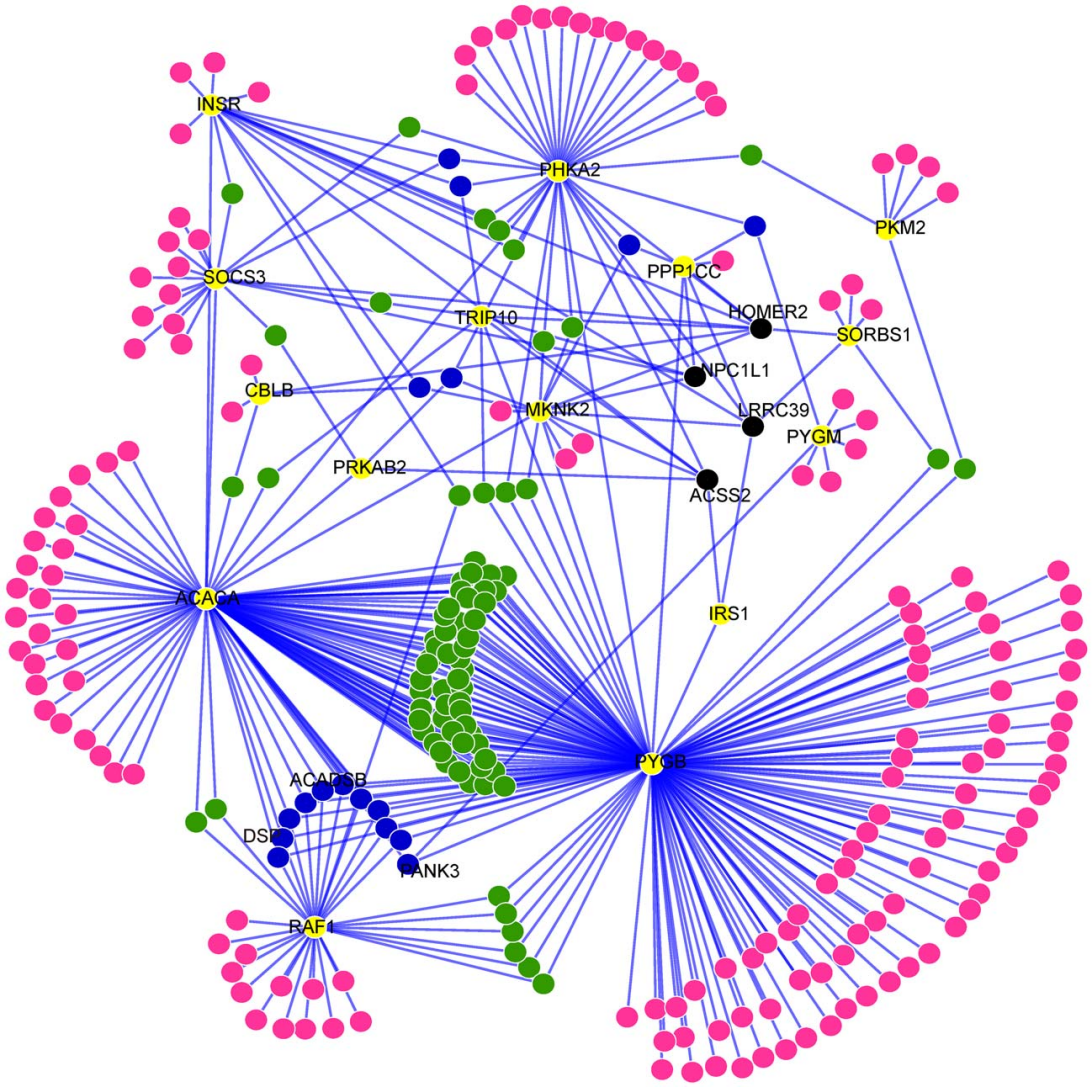
Sub-network for insulin signaling genes and their neighbor genes in the synergy network. Yellow nodes represent insulin signaling genes, and pink, green, blue and black nodes represent the neighbor genes connecting to one, two, three and more insulin signaling genes, respectively. The fifteen insulin genes, the top three neighbor genes (in Table 2) and the four genes connecting to more than three genes were labeled with gene names for reference.

We evaluated the biological relevance of insulin signaling, the most significantly enriched pathway, and its neighbor genes. In the synergy network, there are a total of 262 neighbor genes (see Table S3) connected to the insulin signaling pathway (Figure 2 and Figure S4), and most of these neighbor genes (more than 90%) are connected to one or two insulin signaling genes. We focused on only those neighbor genes that are connected to 3 or more insulin signaling genes in the synergy network. For each of these neighbor genes, the statistical significance of its association with the insulin signaling pathway was evaluated by the hyper-geometric test. The neighbor genes significantly associated with insulin signaling are listed and ranked according to their p-values in Table 2.

**Table 2:** Neighbors significantly associated with insulin signaling pathway.

Gene	Connections to insulin signaling	Degree (overall network)	p-value	Association with insulin signaling
DSP	3	12	4.18E-03	Unknown
ACADSB	3	12	4.18E-03	Unknown^1^
PANK3	3	15	7.50E-03	Known, [69]
SLC39A3	3	18	9.60E-03	Known, [70], [71], [72]
ICA1	3	20	1.24E-02	Known, [73]
TTYH1	3	30	2.94E-02	Known, [74]
EP400NL	3	31	2.94E-02	Unknown
EGFR	3	36	3.59E-02	Known, [75], [76]
C6orf150	3	37	3.59E-02	Unknown
FAM69B	3	37	3.59E-02	Unknown
HOMER2	8	180	4.84E-02	Known, [77], [78]

1 **ACADSB** is involved in the metabolism of fatty acids and branch chained amino acids [79]. Prolonged treatment with long chain FFAs, including palmitate, increases FA oxidation [12], [80], which has been proposed to serve as a protective mechanism against the potential toxic effects of long chain fatty acids [80], [81]. Insulin, on the other hand, down-regulates FA oxidation in various cell types [82], [83]. Since palmitate can impair the insulin signaling pathway [84], it is reasonable that ACADSB emerges as a top neighbor of the insulin signaling pathway.

### 4 DSP: the top neighbor

**Figure 3:**
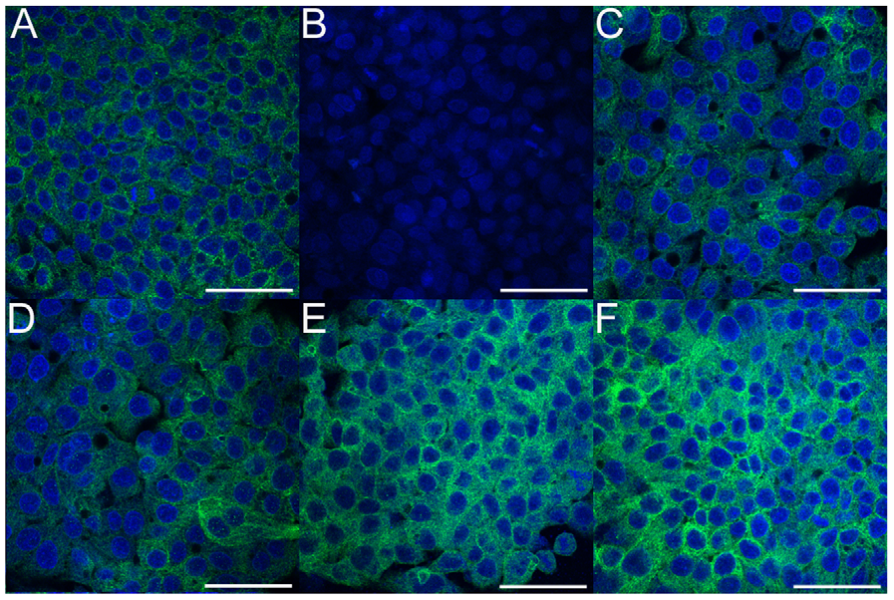
Immuno-fluorescence images of HepG2 cells stained for DSP (green) and cell nuclei (blue) obtained using confocal microscopy (see methods). Scale bars represent 50 µm. A) Untreated, control cells grown in regular growth media. B) Cells treated with palmitate for 48 hours show decrease in DSP levels C) Cells treated with palmitate for 48 hours and recovered in normal growth media for 72 hours show partial recovery of DSP expression. Cells treated with palmitate for 48 hours and recovered in growth media with insulin for D) 24 hours and E) 48 hours show partial recovery, whereas for F) 72 hours show complete recovery of DSP expression.

**Figure 4:**
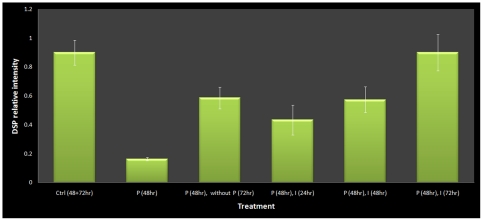
Quantitative effects of palmitate and insulin treatment on DSP expression (see methods). Bars indicate relative expression of DSP under various treatment conditions. Palmitate treatment significantly decreases DSP expression where as subsequent treatment with insulin restores DSP expression. Lines represent pairs of condition where changes in DSP level are significant. * indicates significant difference (p<0.05) from control cells. ** indicates significant difference (p<0.05) from palmitate (48 hours) treated cells.

The junction protein, DSP, was recovered as the top-neighbor of insulin signaling pathway from the synergy network. Loss of DSP expression has been reported in more severe forms of liver disease, i.e. cirrhosis and HCC, but this has not been implicated in the pathology of fatty liver disease. Hence, we investigated the effects of palmitate treatment on the expression of DSP in HepG2 cells. Since, DSP was recovered as a neighbor of the insulin signaling pathway, we also studied the effect of insulin treatment on DSP expression. HepG2 cells were grown to confluency and treated with palmitate containing media. We observed cellular expression of DSP by measuring the fluorescence levels of DSP with immunostaining and confocal microscopy (Figure 3). Relative intensity of DSP against nuclear staining was used to quantify and normalize the amount of DSP under each treatment condition (Figure 4). Finally, we compared changes in relative DSP expression between treatment conditions and determined statistically significant pairs using Tukey's HSD post-hoc analysis (Figure 4).

### Palmitate reduces DSP expression

First, we evaluated the effect of palmitate treatment on DSP expression. We treated HepG2 cells with 0.7 mM palmitate for 48 hours and observed the fluorescence levels of DSP (Figure 3B). When compared to control (Figure 3A), we found that palmitate treated cells showed a significant decrease in DSP expression levels (p<0.05).

### Insulin enhances recovery of DSP expression

Next, we examined the recovery of DSP expression in palmitate treated cells by removing palmitate after 48 hours of treatment and adding regular growth medium. The cells were allowed to grow for another 72 hours (Figure 3C) and showed some recovery of DSP expression with time, albeit statistically different from control (p<0.05). To assess the effect of insulin on the recovery of DSP expression, we treated HepG2 cells with physiological concentration of insulin (1 nM) [32] for up to 72 hours, following the removal of palmitate after 48 hours of treatment. Fluorescence levels of DSP in the cells were analyzed every 24 hours. As seen in Figure 3D–E, cells treated with insulin for 24 and 48 hours show partial recovery of DSP levels compared to palmitate treated cells (p<0.05), but cells treated with insulin for 72 hours show a prominent increase in DSP levels (p<0.05) (Figure 3F). In fact, the levels of DSP after 72 hours of insulin treatment are statistically similar to the levels in the control cells (p>0.05). The results suggest the expression of DSP that was lost upon palmitate treatment recovered to basal levels by treating the cells with insulin. Although, cells did recover some expression of DSP after removal of palmitate from the medium, the recovery was not restored to basal levels.

Thus, our experiment supports the association of the palmitate-induced cytotoxic phenotype with the DSP-insulin signaling pathway in our network. Namely, that DSP and insulin signaling act synergistically to ensure the proper function of adherence junctions and the lost or reduction in DSP expression and insulin signaling by palmitate treatment contributes to the cytotoxic effect of palmitate. Thus suggesting that in liver cells DSP may be a novel target of palmitate-induced cytotoxicity.

### 5 Differential correlation analysis fails to identify DSP

Extant methods based on differential correlation have been used to uncover phenotype-specific gene interactions [33]. However, the quality of the data (i.e. sample size or noise level) would affect the results obtained, i.e. in the correlation coefficients calculated, making it difficult to determine whether the difference in the correlation across the phenotypes are real changes or simply an artifact due to the size or noise level. In contrast, the information synergy approach takes the phenotype data directly into account, thus the influence of the difference in the data quality across phenotypes is less of a concern than in the correlation-based methods. We compared the performance of information synergy approach to a standard differential correlation approach [25] for reconstructing phenotype-specific networks. The statistics for this comparison are shown in Table 3. As seen in Table 3, the insulin signaling pathway was recovered as the top pathway in the differential correlation network. But, DSP was not found to be significantly associated with the insulin signaling pathway.

**Table 3:** The statistics of the two networks and association b/w DSP and insulin signaling.

		Synergy network	Differential correlation network
Statistics for whole network	# genes in the network	570	610
	# connections in the network	4376	87054
	# insulin signaling genes in the network	15	15
	Rank of insulin signaling in term of statistical significance in the network	1st	1st
Statistics for DSP	Degree of DSP in the network	12	210
	# insulin signaling genes connecting to DSP	3	3
	FDR adjusted p-value of the association b/w DSP and insulin signaling	4.18E-3	0.882
	Rank of DSP among the genes connected to at least three insulin signaling genes based on statistical significance	1st	549th

**Figure 5:**
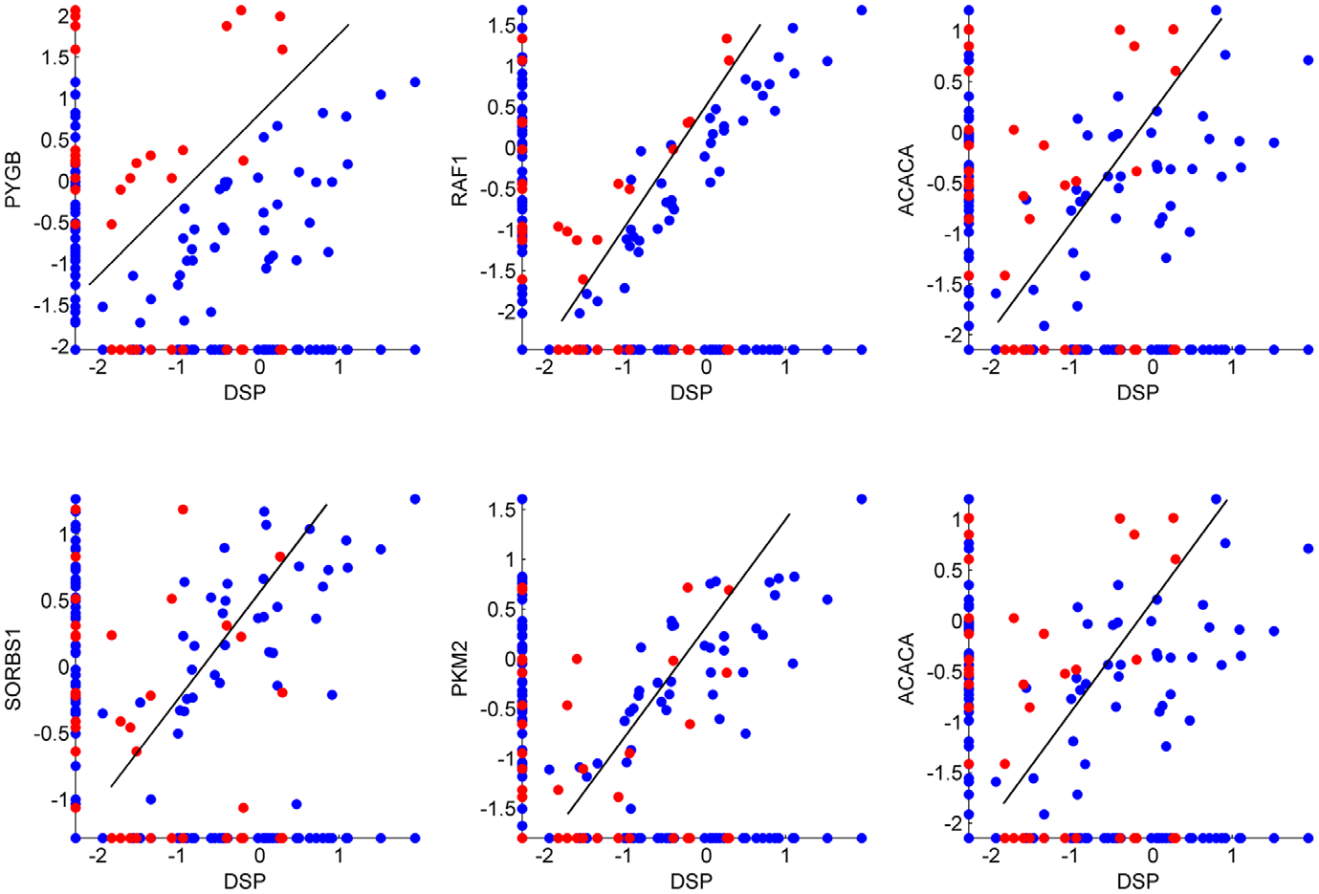
The expressions of gene pairs, namely of insulin signaling genes and DSP. Shown are three pairs of insulin signaling genes connected to DSP in the synergy analysis (top) and three pairs obtained from the differential correlation analysis (bottom). Red and blue points represent toxic (treated with palmitate) and nontoxic samples, respectively.

We further investigated the ability of genes paired with DSP to distinguish the two phenotypes. The joint expressions of these gene pairs are shown in Figure 5. One gene (ACACA) was identified by both synergy analysis and differential correlation. From Figure 5, it is evident that the two phenotypes are distinctly separated in the synergy analysis plots whereas the phenotypes in the correlation analysis plots do not separate distinctly. Thus, the synergy analysis was more effective in identifying gene pairs that can distinguish between phenotypes.

## Discussion

We presented an integrative methodology to reconstruct a gene network that demonstrates cooperativity plays an important role in complex diseases. There are three major contributions from this analysis. First, we presented the idea of information synergy and applied this method to microarray data from palmitate treated HepG2 cells: a condition relevant to fatty liver disease. Second, we showed that DSP is a novel target of palmitate treatment. Third, we showed that insulin impacts DSP expression, and palmitate, known to antagonize insulin signaling, also negatively impacts DSP, contributing to the cytotoxic effect of palmitate. Overall, we suggest that a synergy network-based gene and pathway association analysis has the potential to reveal novel targets or mechanisms underlying biological processes, exemplified by the uncovering of DSP as a novel player in palmitate-induced cytoxicity.

The concept of synergy is intuitive and relevant to biological systems. For example, two transcription factors may play a cooperative role in determining the expression of a common gene. Similarly, interactions may occur between signaling proteins, i.e. cross-talk, and such associations may also be seen in receptor mediated transduction. Information synergy aims to capture cooperative effects, and in this study, on the impact of these effects on the induction a particular phenotype. There are several examples of synergistic interactions implicated in the pathology of fatty liver disease. For example, Smith J.J et al. showed that two groups of transcription factors worked in a combinatorial manner to control the transcriptional responses to fatty acids [34], thus supporting that cooperativity is important in regulating cellular processes. Similarly, co-expression of HNF4 and GATA transcription factor could synergistically activate the expressions of two genes involved in cholesterol homeostasis, and their binding sites are essential for maximal synergism [35]. Peroxisome proliferator activated receptors (PPARs), one of the best characterized nuclear receptors that mediate transcriptional activities of long-chain unsaturated fatty acids, was found to selectively cooperate with fatty acid binding proteins (FABPs) to regulate transcription [36]. Sterol receptor element-binding protein (SREBP-1c), a gatekeeper of lipotoxicity [37], functions together with BETA2, LXRs and SP1, to regulate various cellular processes, such as insulin signaling [38], lipid synthesis [39], and cholesterol biosynthesis [40]. It is evident from these examples that identifying genes with cooperative effects [13], instead of limiting efforts to individual genes, has the potential to reveal novel genes or interactions, and ultimately help elucidate the mechanism of progression of complex diseases such as NAFLD. Along this line, differential correlation based approaches have been applied to identify linear relationships between genes and phenotype. However, information synergy has the distinct advantage in their ability to capture gene pairs with different types of dependency (not limited to linear correlation), as long as the gene pairs provide additional information on the phenotype. This makes it particularly appealing for complex scenarios such as uncovering gene cooperation in the induction of a phenotype or disease, where the interactions may not be linear. Thus, information synergy is a promising approach for constructing phenotype-specific networks, and provides a complementary approach to correlation-based methods.

Although synergistic gene pairs have been shown valuable in discriminating phenotypes, interpreting the roles of the individual gene pairs remains a challenge due to the lack of sufficient functional or structural annotations for many of the genes, thereby formulating plausible hypotheses of the gene pairs more difficult. In contrast, organizing the individual gene pairs into a network (synergy network) and then performing gene module level analysis [41], i.e. identifying the pathways over-represented in network or pathway-gene associations, could help to hint at potential mechanisms as shown in our study. Nevertheless, investigating individual gene pairs would still be valuable in the future. Since the gene pairs identified from the microarray data do not necessarily interact directly with each other, future investigation of individual gene pairs coupled with physical interaction data (i.e. protein-protein and protein-DNA interaction) should further improve the analysis.

The application of information synergy methodology to microarray data obtained from FFA-treated HepG2 cells revealed insulin signaling as the most significantly enriched biological pathway. Thus, our analysis successfully recovered insulin signaling as a hallmark characteristic pathway involved in fatty liver disease [42]. We further examined and confirmed the effects of palmitate treatment on DSP, the top synergistic neighbor of insulin signaling pathway.

Through synergy analysis we found that expression of DSP is reduced by palmitate treatment in HepG2 cells. DSP is a desmosomal protein essential for intercellular attachment by desmosomes. In a study of adhesion molecules in HCC, Cao et al. found that moderately differentiated hepatocytes tend to show a reduction in DSP protein expression, whereas poorly differentiated hepatocytes show complete loss of DSP protein expression [43]. It has been suggested that DSP expression levels are inversely correlated with cell growth and division. As shown in a study with squamous cell carcinoma, inhibition of EGF (epidermal growth factor) using antagonists led to an increase in DSP protein levels [44]. Another study has suggested that DSP may play a role in tumor-matrix interaction as it was found to be a major component of exosomes secreted by mesothelioma cells [45]. Other studies have also suggested that loss of desmosomes may be associated with epithelial to mesenchymal transition (EMT) by means which hepatocytes may acquire malignant characteristics [46]. Although, such associations were reported in HCC, our results suggest that the impact on DSP may be an early event in the pathogenesis of fatty liver disease. In other words, reduced expression of DSP may contribute to the progression to more severe forms of liver diseases.

From Table 1 adherens junction was identified as a highly enriched category in our synergy network. Treatment with palmitate has been shown to affect cellular adherence and cytoskeletal structure in hepatocytes [29], [30], although the exact mechanism and whether DSP is involved in mediating these processes is unknown. Since, keratins require DSP at the desmosomal plaque for anchoring to the cell membrane [17], our results open up the possibility that loss of cytoskeletal structure may be associated with the loss of DSP.

Previous studies have shown that inherited mutations in DSP are a characteristic feature of a number diseases of the skin and heart including, skin fragility or wooly hair syndrome [47], arrhythmogenic right ventricular dysplasia [17], [48], lethal acantholytic epidermolysis bullosa [49], and dilated cardiomyopathy with wooly hair and keratoderma [50]. It is notable that in recent years some research groups have suggested the risk of cardiovascular disease (CVD) may be elevated in NAFLD patients [51], [52], [53]. Given that alterations in DSP expression are associated with heart disease and since DSP levels are reduced by palmitate treatment, it raises the possibility that DSP may serve as a potential link between fatty liver disease and heart disease.

We also explored the relationship between DSP and insulin signaling and found that insulin treatment enhances the recovery of DSP expression lost due to palmitate treatment. It is known that palmitate can induce resistance to insulin signaling in hepatocytes [15] and several mechanisms have been proposed to explain this observation. Studies have suggested that PKCA (Protein Kinase C, alpha isoform), known to be activated by lipids (diacylglycerols) [54], shares an antagonistic relationship with insulin signaling, and that knock-out of PKCA enhances insulin signaling [55]. Interestingly, the activation of PKCA also affects the dynamics of desmosomal complex at plasma membrane [56], [57]. Junction plakoglobin co-localizes with DSP at the desmosomal plaque. It has been shown in a study with mice cardiomyocytes that inhibition of DSP expression leads to change in localization of plakoglobins from cytoplasm to nucleus. This was shown to antagonize Wnt/β-catenin signaling pathway, and trigger the accumulation of fat droplets [58]. It is noteworthy that Wnt signaling is a significantly enriched pathway in the synergy network (See Table 1). Wnt signaling is frequently attenuated in HCC patients and is associated with poor prognosis [59]. Furthermore, recent studies have confirmed that activation of Wnt signaling enhances insulin sensitivity [60], [61]. This suggests that palmitate induced loss of DSP protein can further enhance insulin resistance through the Wnt/β-catenin signaling pathway. Thus, it may not be a coincidence that DSP was recovered as a synergistic pair to the insulin signaling pathway in the network.

According to one estimate, about one-third of the general population in the United States suffers from NAFLD [62]. Follow up studies of patients suffering from this burgeoning disease have shown that their survival is significantly lower than that of the general population [63]. Furthermore, NAFLD can progress to the more severe steatohepatitis (NASH) in a number of patients, which may further develop into cirrhosis and eventually into liver carcinoma (HCC) [64]. Unfortunately, the factors responsible for the pathology and progression of liver disease in these patients remain poorly understood. Our studies confirmed a potential link between palmitate, insulin signaling and DSP, and are currently further investigating the mechanisms contributing towards progression to more severe forms of liver disease.

## Supporting Information

Figure S1 Four Representative Trends of the Metabolites. Eleven metabolites differed significantly across the three conditions (treated by BSA, Palmitate and Oleate), and four representative trends were extracted from these metabolites. Trend I: BSA < Palm and Palm > Ole; Trend II: BSA > Palm and Palm < Ole; Trend III: BSA < Palm < Ole; Trend IV: BSA > Palm > Ole.
(TIFF)

Figure S2 Degree distribution of synergy network and Random network. The random network was generated based on Erdös-Rényi model, with same number of nodes and edges as synergy network. The degree distribution in synergy network is clearly different with that in random network.
(TIF)

Figure S3 Different types of correlation patterns captured by synergy analysis. Blue and red points represent nontoxic and toxic samples, respectively. These patterns were specified in [30], and their definitions were given below: (1) Gap: gene positively correlated; phenotype associated with the difference of gene expression; Substitute: gene negatively correlated; phenotype associated with the sum of gene expression; (3)On/off: turning on or off both genes lead to same phenotype.
(TIF)

Figure S4 Subnetwork for insulin signaling pathway (all nodes labeled with gene symbols).
(TIF)

Table S1 The list of 610 genes selected for synergy analysis.
(DOC)

Table S2 All KEGG pathways significantly enriched in the synergy network.
(DOC)

Table S3 All neighbor genes significantly associated with insulin signaling.
(DOC)

## References

[1] Donnelly KL, Smith CI, Schwarzenberg SJ, Jessurun J, Boldt MD (2005). Sources of fatty acids stored in liver and secreted via lipoproteins in patients with nonalcoholic fatty liver disease.. J Clin Invest.

[2] Angulo P (2007). Obesity and nonalcoholic fatty liver disease.. Nutr Rev.

[3] Musso G, Gambino R, De Michieli F, Cassader M, Rizzetto M (2003). Dietary habits and their relations to insulin resistance and postprandial lipemia in nonalcoholic steatohepatitis.. Hepatology.

[4] Zelber-Sagi S, Nitzan-Kaluski D, Goldsmith R, Webb M, Blendis L (2007). Long term nutritional intake and the risk for non-alcoholic fatty liver disease (NAFLD): a population based study.. J Hepatol.

[5] Musso G, Gambino R, Cassader M (2009). Recent insights into hepatic lipid metabolism in non-alcoholic fatty liver disease (NAFLD).. Prog Lipid Res.

[6] Li Z, Srivastava S, Mittal S, Yang X, Sheng L (2007). A Three Stage Integrative Pathway Search (TIPS) framework to identify toxicity relevant genes and pathways.. BMC Bioinformatics.

[7] Li Z, Srivastava S, Yang X, Mittal S, Norton P (2007). A hierarchical approach employing metabolic and gene expression profiles to identify the pathways that confer cytotoxicity in HepG2 cells.. BMC Syst Biol.

[8] Srivastava S, Li Z, Yang X, Yedwabnick M, Shaw S (2007). Identification of genes that regulate multiple cellular processes/responses in the context of lipotoxicity to hepatoma cells.. BMC Genomics.

[9] Yang X, Zhou Y, Jin R, Chan C (2009). Reconstruct modular phenotype-specific gene networks by knowledge-driven matrix factorization.. Bioinformatics.

[10] Li Z, Srivastava S, Findlan R, Chan C (2008). Using dynamic gene module map analysis to identify targets that modulate free fatty acid induced cytotoxicity.. Biotechnol Prog.

[11] Remenyi A, Scholer HR, Wilmanns M (2004). Combinatorial control of gene expression.. Nat Struct Mol Biol.

[12] Srivastava S, Chan C (2008). Application of metabolic flux analysis to identify the mechanisms of free fatty acid toxicity to human hepatoma cell line.. Biotechnol Bioeng.

[13] Anastassiou D (2007). Computational analysis of the synergy among multiple interacting genes.. Mol Syst Biol.

[14] Bugianesi E, McCullough AJ, Marchesini G (2005). Insulin resistance: a metabolic pathway to chronic liver disease.. Hepatology.

[15] Ruddock MW, Stein A, Landaker E, Park J, Cooksey RC (2008). Saturated fatty acids inhibit hepatic insulin action by modulating insulin receptor expression and post-receptor signalling.. J Biochem.

[16] Getsios S, Huen AC, Green KJ (2004). Working out the strength and flexibility of desmosomes.. Nat Rev Mol Cell Biol.

[17] Rampazzo A, Nava A, Malacrida S, Beffagna G, Bauce B (2002). Mutation in human desmoplakin domain binding to plakoglobin causes a dominant form of arrhythmogenic right ventricular cardiomyopathy.. Am J Hum Genet.

[18] Vinken M, Papeleu P, Snykers S, De Rop E, Henkens T (2006). Involvement of cell junctions in hepatocyte culture functionality.. Crit Rev Toxicol.

[19] Cao Y, Chang H, Li L, Cheng RC, Fan XN (2007). Alteration of adhesion molecule expression and cellular polarity in hepatocellular carcinoma.. Histopathology.

[20] Barrett T, Troup DB, Wilhite SE, Ledoux P, Evangelista C (2011). NCBI GEO: archive for functional genomics data sets—10 years on.. Nucleic Acids Res.

[21] Yang X, Wang X, Wu M, Dalkic E, Chan C, Jayaraman A, Hahn J (2009). Construction of the gene network by synergy analysis of the genes related to palmitate-induced cytotoxicity.. Methods in Bioengineering in Systems Analysis of Biological Networks: Artech House Publishers.

[22] Watkinson J, Wang X, Zheng T, Anastassiou D (2008). Identification of gene interactions associated with disease from gene expression data using synergy networks.. BMC Syst Biol.

[23] Benjamini Y, Hochberg Y (1995). Controlling the False Discovery Rate - a Practical and Powerful Approach to Multiple Testing.. Journal of the Royal Statistical Society Series B-Methodological.

[24] Carmona-Saez P, Chagoyen M, Tirado F, Carazo JM, Pascual-Montano A (2007). GENECODIS: a web-based tool for finding significant concurrent annotations in gene lists.. Genome Biol.

[25] Zhang J, Li J, Deng H (2008). Class-specific correlations of gene expressions: identification and their effects on clustering analyses.. Am J Hum Genet.

[26] Dettling M, Gabrielson E, Parmigiani G (2005). Searching for differentially expressed gene combinations.. Genome Biol.

[27] Guo W, Wong S, Xie W, Lei T, Luo Z (2007). Palmitate modulates intracellular signaling, induces endoplasmic reticulum stress, and causes apoptosis in mouse 3T3-L1 and rat primary preadipocytes.. Am J Physiol Endocrinol Metab.

[28] Zhou Q, Du J, Hu Z, Walsh K, Wang XH (2007). Evidence for adipose-muscle cross talk: opposing regulation of muscle proteolysis by adiponectin and Fatty acids.. Endocrinology.

[29] Draghici S, Khatri P, Tarca AL, Amin K, Done A (2007). A systems biology approach for pathway level analysis.. Genome Res.

[30] Swagell CD, Henly DC, Morris CP (2005). Expression analysis of a human hepatic cell line in response to palmitate.. Biochem Biophys Res Commun.

[31] Tsang MY, Cowie SE, Rabkin SW (2004). Palmitate increases nitric oxide synthase activity that is involved in palmitate-induced cell death in cardiomyocytes.. Nitric Oxide.

[32] Yang X, Nath A, Opperman MJ, Chan C (2010). The double-stranded RNA-dependent protein kinase differentially regulates insulin receptor substrates 1 and 2 in HepG2 cells.. Mol Biol Cell.

[33] de la Fuente A (2010). From ‘differential expression’ to ‘differential networking’ - identification of dysfunctional regulatory networks in diseases.. Trends Genet.

[34] Smith JJ, Ramsey SA, Marelli M, Marzolf B, Hwang D (2007). Transcriptional responses to fatty acid are coordinated by combinatorial control.. Mol Syst Biol.

[35] Sumi K, Tanaka T, Uchida A, Magoori K, Urashima Y (2007). Cooperative interaction between hepatocyte nuclear factor 4 alpha and GATA transcription factors regulates ATP-binding cassette sterol transporters ABCG5 and ABCG8.. Mol Cell Biol.

[36] Tan NS, Shaw NS, Vinckenbosch N, Liu P, Yasmin R (2002). Selective cooperation between fatty acid binding proteins and peroxisome proliferator-activated receptors in regulating transcription.. Mol Cell Biol.

[37] Slawik M, Vidal-Puig AJ (2006). Lipotoxicity, overnutrition and energy metabolism in aging.. Ageing Res Rev.

[38] Amemiya-Kudo M, Oka J, Ide T, Matsuzaka T, Sone H (2005). Sterol regulatory element-binding proteins activate insulin gene promoter directly and indirectly through synergy with BETA2/E47.. J Biol Chem.

[39] Higuchi N, Kato M, Shundo Y, Tajiri H, Tanaka M (2008). Liver X receptor in cooperation with SREBP-1c is a major lipid synthesis regulator in nonalcoholic fatty liver disease.. Hepatol Res.

[40] Reed BD, Charos AE, Szekely AM, Weissman SM, Snyder M (2008). Genome-wide occupancy of SREBP1 and its partners NFY and SP1 reveals novel functional roles and combinatorial regulation of distinct classes of genes.. PLoS Genet.

[41] Wang X, Dalkic E, Wu M, Chan C (2008). Gene module level analysis: identification to networks and dynamics.. Curr Opin Biotechnol.

[42] Mattar SG, Velcu LM, Rabinovitz M, Demetris AJ, Krasinskas AM (2005). Surgically-induced weight loss significantly improves nonalcoholic fatty liver disease and the metabolic syndrome.. Ann Surg.

[43] Han SF, Deng RL, Xu HR, Cao YF, Wang XY (2007). [Photosynthesis and active-oxygen-scavenging enzyme activities in rice varieties with different phosphorus efficiency under phosphorus stress].. Ying Yong Sheng Tai Xue Bao.

[44] Lorch JH, Klessner J, Park JK, Getsios S, Wu YL (2004). Epidermal growth factor receptor inhibition promotes desmosome assembly and strengthens intercellular adhesion in squamous cell carcinoma cells.. J Biol Chem.

[45] Hegmans JP, Bard MP, Hemmes A, Luider TM, Kleijmeer MJ (2004). Proteomic analysis of exosomes secreted by human mesothelioma cells.. Am J Pathol.

[46] Chidgey M, Dawson C (2007). Desmosomes: a role in cancer?. Br J Cancer.

[47] Whittock NV, Wan H, Morley SM, Garzon MC, Kristal L (2002). Compound heterozygosity for non-sense and mis-sense mutations in desmoplakin underlies skin fragility/woolly hair syndrome.. J Invest Dermatol.

[48] Yang Z, Bowles NE, Scherer SE, Taylor MD, Kearney DL (2006). Desmosomal dysfunction due to mutations in desmoplakin causes arrhythmogenic right ventricular dysplasia/cardiomyopathy.. Circ Res.

[49] Jonkman MF, Pasmooij AM, Pasmans SG, van den Berg MP, Ter Horst HJ (2005). Loss of desmoplakin tail causes lethal acantholytic epidermolysis bullosa.. Am J Hum Genet.

[50] Uzumcu A, Norgett EE, Dindar A, Uyguner O, Nisli K (2006). Loss of desmoplakin isoform I causes early onset cardiomyopathy and heart failure in a Naxos-like syndrome.. J Med Genet.

[51] Targher G, Marra F, Marchesini G (2008). Increased risk of cardiovascular disease in non-alcoholic fatty liver disease: causal effect or epiphenomenon?. Diabetologia.

[52] Targher G, Bertolini L, Padovani R, Rodella S, Zoppini G (2010). Prevalence of non-alcoholic fatty liver disease and its association with cardiovascular disease in patients with type 1 diabetes.. J Hepatol.

[53] Edens MA, Kuipers F, Stolk RP (2009). Non-alcoholic fatty liver disease is associated with cardiovascular disease risk markers.. Obes Rev.

[54] Ekinci FJ, Shea TB (1999). Free PKC catalytic subunits (PKM) phosphorylate tau via a pathway distinct from that utilized by intact PKC.. Brain Res.

[55] Leitges M, Plomann M, Standaert ML, Bandyopadhyay G, Sajan MP (2002). Knockout of PKC alpha enhances insulin signaling through PI3K.. Mol Endocrinol.

[56] Hobbs RP, Amargo EV, Somasundaram A, Simpson CL, Prakriya M (2010). The calcium ATPase SERCA2 regulates desmoplakin dynamics and intercellular adhesive strength through modulation of PKC{alpha} signaling..

[57] Bass-Zubek AE, Hobbs RP, Amargo EV, Garcia NJ, Hsieh SN (2008). Plakophilin 2: a critical scaffold for PKC alpha that regulates intercellular junction assembly.. J Cell Biol.

[58] Garcia-Gras E, Lombardi R, Giocondo MJ, Willerson JT, Schneider MD (2006). Suppression of canonical Wnt/beta-catenin signaling by nuclear plakoglobin recapitulates phenotype of arrhythmogenic right ventricular cardiomyopathy.. J Clin Invest.

[59] Polakis P (2000). Wnt signaling and cancer.. Genes Dev.

[60] Abiola M, Favier M, Christodoulou-Vafeiadou E, Pichard AL, Martelly I (2009). Activation of Wnt/beta-catenin signaling increases insulin sensitivity through a reciprocal regulation of Wnt10b and SREBP-1c in skeletal muscle cells.. PLoS One.

[61] Yoon JC, Ng A, Kim BH, Bianco A, Xavier RJ (2010). Wnt signaling regulates mitochondrial physiology and insulin sensitivity.. Genes Dev.

[62] Browning JD, Szczepaniak LS, Dobbins R, Nuremberg P, Horton JD (2004). Prevalence of hepatic steatosis in an urban population in the United States: impact of ethnicity.. Hepatology.

[63] Ekstedt M, Franzen LE, Mathiesen UL, Thorelius L, Holmqvist M (2006). Long-term follow-up of patients with NAFLD and elevated liver enzymes.. Hepatology.

[64] de Alwis NM, Day CP (2008). Non-alcoholic fatty liver disease: the mist gradually clears.. J Hepatol.

[65] Smith J, Su X, El-Maghrabi R, Stahl PD, Abumrad NA (2008). Opposite regulation of CD36 ubiquitination by fatty acids and insulin: effects on fatty acid uptake.. J Biol Chem.

[66] Gao D, Nong S, Huang X, Lu Y, Zhao H (2010). The effects of palmitate on hepatic insulin resistance are mediated by NADPH Oxidase 3-derived reactive oxygen species through JNK and p38MAPK pathways.. J Biol Chem.

[67] Franch-Marro X, Wendler F, Griffith J, Maurice MM, Vincent JP (2008). In vivo role of lipid adducts on Wingless.. J Cell Sci.

[68] Borradaile NM, Buhman KK, Listenberger LL, Magee CJ, Morimoto ET (2006). A critical role for eukaryotic elongation factor 1A-1 in lipotoxic cell death.. Mol Biol Cell.

[69] Leonardi R, Zhang YM, Rock CO, Jackowski S (2005). Coenzyme A: back in action.. Prog Lipid Res.

[70] Huang L, Yan M, Kirschke CP (2010). Over-expression of ZnT7 increases insulin synthesis and secretion in pancreatic beta-cells by promoting insulin gene transcription..

[71] Nicolson TJ, Bellomo EA, Wijesekara N, Loder MK, Baldwin JM (2009). Insulin storage and glucose homeostasis in mice null for the granule zinc transporter ZnT8 and studies of the type 2 diabetes-associated variants.. Diabetes.

[72] Smidt K, Jessen N, Petersen AB, Larsen A, Magnusson N (2009). SLC30A3 responds to glucose- and zinc variations in beta-cells and is critical for insulin production and in vivo glucose-metabolism during beta-cell stress.. PLoS One.

[73] Buffa L, Fuchs E, Pietropaolo M, Barr F, Solimena M (2008). ICA69 is a novel Rab2 effector regulating ER-Golgi trafficking in insulinoma cells.. Eur J Cell Biol.

[74] Anderson AA, Helmering J, Juan T, Li CM, McCormick J (2009). Pancreatic islet expression profiling in diabetes-prone C57BLKS/J mice reveals transcriptional differences contributed by DBA loci, including Plagl1 and Nnt.. Pathogenetics.

[75] Prada PO, Ropelle ER, Mourao RH, de Souza CT, Pauli JR (2009). EGFR tyrosine kinase inhibitor (PD153035) improves glucose tolerance and insulin action in high-fat diet-fed mice.. Diabetes.

[76] Chong MP, Barritt GJ, Crouch MF (2004). Insulin potentiates EGFR activation and signaling in fibroblasts.. Biochem Biophys Res Commun.

[77] Shiraishi Y, Mizutani A, Yuasa S, Mikoshiba K, Furuichi T (2004). Differential expression of Homer family proteins in the developing mouse brain.. J Comp Neurol.

[78] Rong R, Ahn JY, Huang H, Nagata E, Kalman D (2003). PI3 kinase enhancer-Homer complex couples mGluRI to PI3 kinase, preventing neuronal apoptosis.. Nat Neurosci.

[79] Arden KC, Viars CS, Fu K, Rozen R (1995). Localization of short/branched chain acyl-CoA dehydrogenase (ACADSB) to human chromosome 10.. Genomics.

[80] Ceddia RB (2005). Direct metabolic regulation in skeletal muscle and fat tissue by leptin: implications for glucose and fatty acids homeostasis.. Int J Obes (Lond).

[81] Kahn BB, Alquier T, Carling D, Hardie DG (2005). AMP-activated protein kinase: ancient energy gauge provides clues to modern understanding of metabolism.. Cell Metab.

[82] Dyck DJ, Steinberg G, Bonen A (2001). Insulin increases FA uptake and esterification but reduces lipid utilization in isolated contracting muscle.. Am J Physiol Endocrinol Metab.

[83] Topping DL, Mayes PA (1972). The immediate effects of insulin and fructose on the metabolism of the perfused liver. Changes in lipoprotein secretion, fatty acid oxidation and esterification, lipogenesis and carbohydrate metabolism.. Biochem J.

[84] Storz P, Doppler H, Wernig A, Pfizenmaier K, Muller G (1999). Cross-talk mechanisms in the development of insulin resistance of skeletal muscle cells palmitate rather than tumour necrosis factor inhibits insulin-dependent protein kinase B (PKB)/Akt stimulation and glucose uptake.. Eur J Biochem.

