# Supplementary figures and images for: Synergy Analysis Reveals Association between Insulin Signaling and Desmoplakin Expression in Palmitate Treated HepG2 Cells
Source: PLoS One. 2011 Nov 23;6(11):e28138. doi: 10.1371/journal.pone.0028138 (PMC3223234; doi:10.1371/journal.pone.0028138)

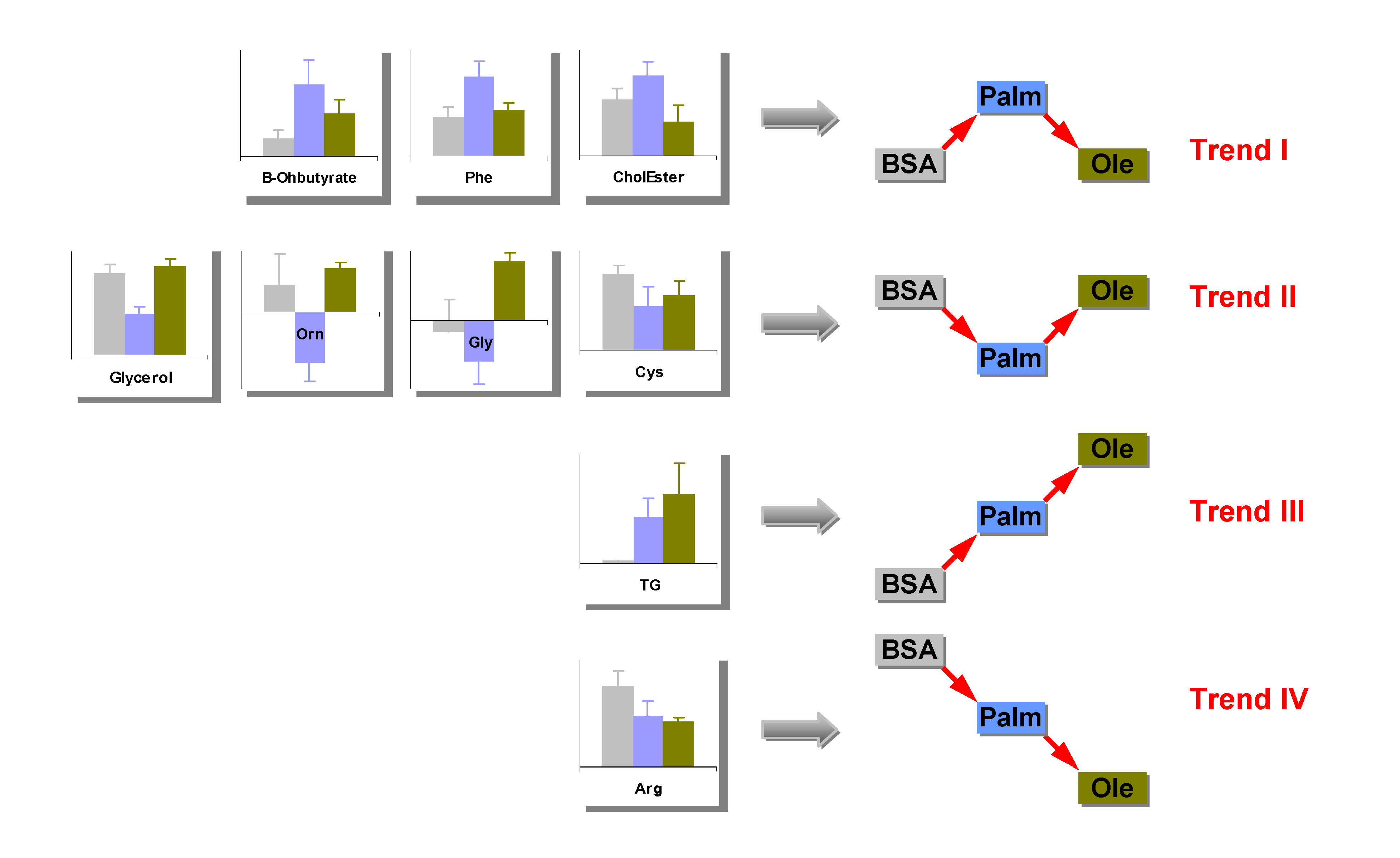

Supplement: Figure S1 — Four Representative Trends of the Metabolites. Eleven metabolites differed significantly across the three conditions (treated by BSA, Palmitate and Oleate), and four representative trends were extracted from these metabolites. Trend I: BSA < Palm and Palm > Ole; Trend II: BSA > Palm and Palm < Ole; Trend III: BSA < Palm < Ole; Trend IV: BSA > Palm > Ole. (TIFF) [file pone.0028138.s001.tif]

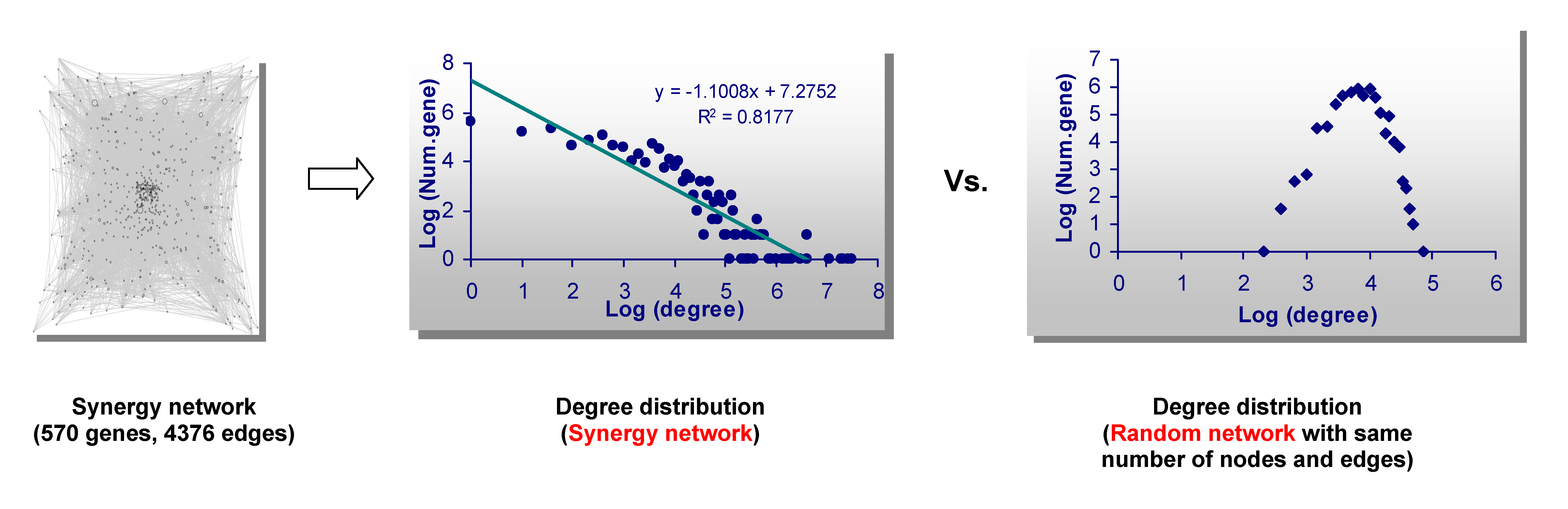

Supplement: Figure S2 — Degree distribution of synergy network and Random network. The random network was generated based on Erdös-Rényi model, with same number of nodes and edges as synergy network. The degree distribution in synergy network is clearly different with that in random network. (TIF) [file pone.0028138.s002.tif]

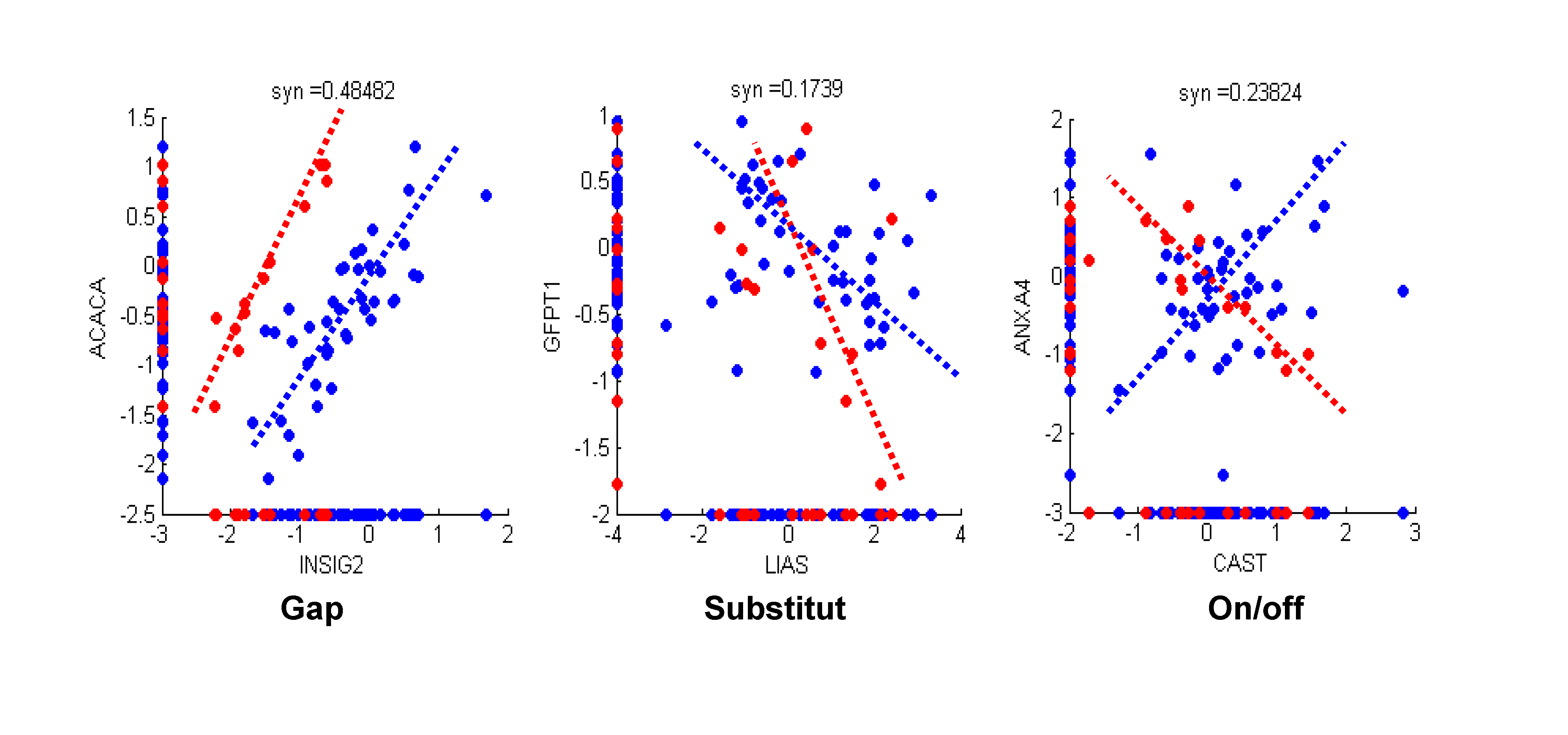

Supplement: Figure S3 — Different types of correlation patterns captured by synergy analysis. Blue and red points represent nontoxic and toxic samples, respectively. These patterns were specified in [30], and their definitions were given below: (1) Gap: gene positively correlated; phenotype associated with the difference of gene expression; Substitute: gene negatively correlated; phenotype associated with the sum of gene expression; (3)On/off: turning on or off both genes lead to same phenotype. (TIF) [file pone.0028138.s003.tif]

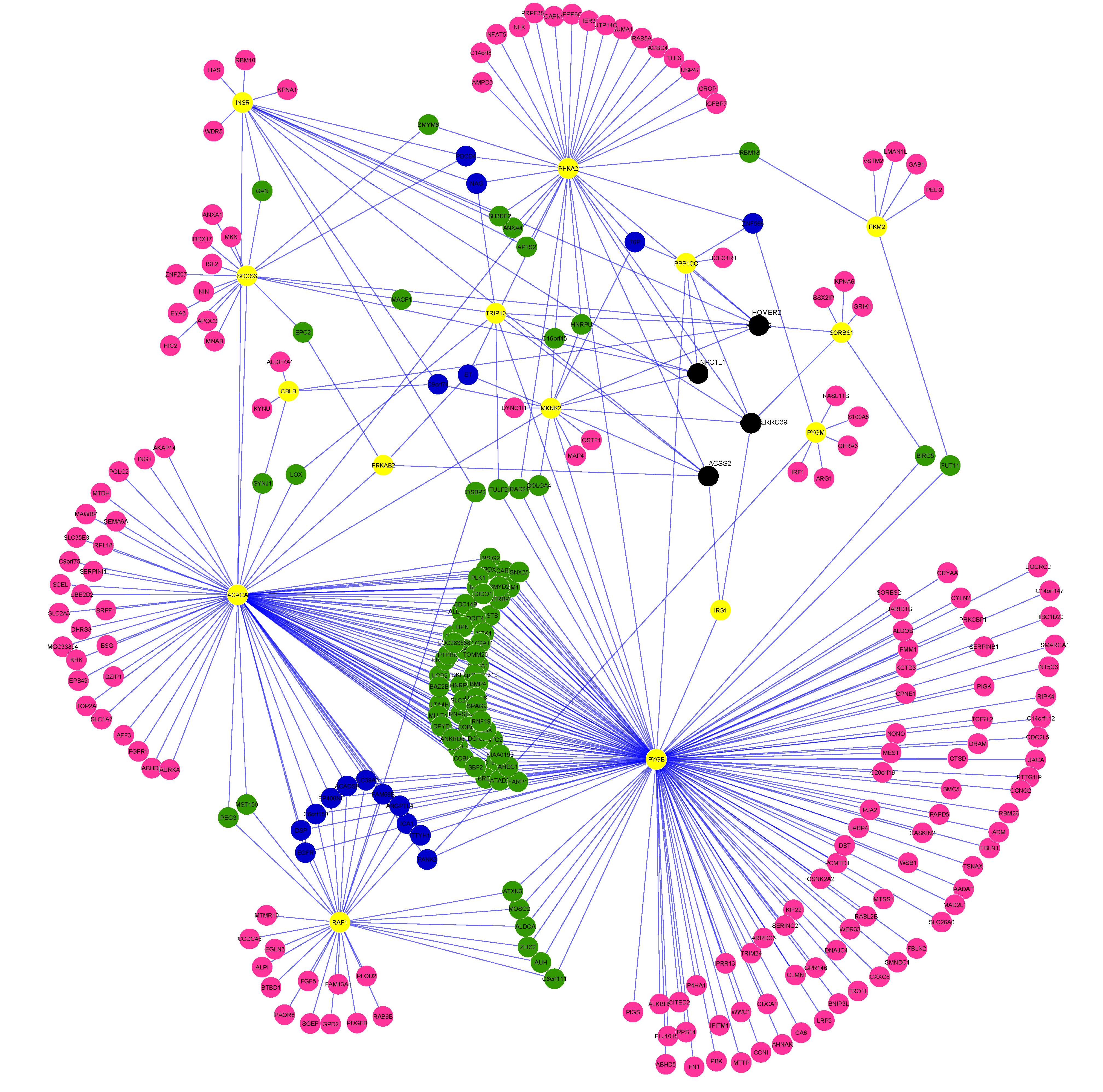

Supplement: Figure S4 — Subnetwork for insulin signaling pathway (all nodes labeled with gene symbols). (TIF) [file pone.0028138.s004.tif]
