# Supplementary material for: Synergy Analysis Reveals Association between Insulin Signaling and Desmoplakin Expression in Palmitate Treated HepG2 Cells
Source: PLoS One. 2011 Nov 23;6(11):e28138. doi: 10.1371/journal.pone.0028138 (PMC3223234; doi:10.1371/journal.pone.0028138)
Supplement: Table S1 — The list of 610 genes selected for synergy analysis. (DOC) [file pone.0028138.s005.doc]

Table S1. List of 610 genes selected for synergy analysis

76P	ARS2	CCT2	DDIT4	FGFR1	HNRPU	LARP4	MTDH
AADAT	ASL	CD3G	DDX17	FGR	HOMER2	LCE1C	MTMR10
ABCA11	ATAD2	CDC14B	DDX6	FKBP7	HOXA13	LDOC1L	MTSS1
ABCB5	ATXN10	CDC2	DGAT1	FLJ10154	HPN	LEAP2	MTTP
ABHD5	ATXN3	CDC2L5	DHFR	FLJ32312	HPS1	LENG4	MUC15
ABHD6	AUH	CDC2L6	DHRS8	FLJ38973	HRASLS3	LGALS3	MXI1
ABI2	AURKA	CDCA1	DHX40	FLNA	HSD17B4	LGR5	MYADM
ACACA	B3GAT2	CEACAM5	DIDO1	FN1	ICA1	LIAS	MYBL2
ACADSB	BAIAP2L1	CEP192	DKFZp762E1312	FNBP4	IER3	LIFR	MYH9
ACBD4	BAZ2B	CEP55	DLC1	FOLR2	IFITM1	LMAN1L	MYO9A
ACMSD	BBS2	CEPT1	DNAJC4	FOS	IGFBP7	LMTK2	NAG
ACOT4	BBX	CETP	DNALI1	FOXO1A	IGSF4	LOC136306	NCK1
ACSS2	BCAP31	CFC1	DNAPTP6	FUT11	IKBKAP	LOC153222	NDUFA10
ADCY1	BIRC5	CHD2	DNM1L	FZD6	IL13RA1	LOC283588	NEK2
ADM	BMP4	CHRDL1	DOCK1	GAB1	IL1B	LOC286161	NEK4
ADSSL1	BNIP3L	CHST10	DPYD	GABRE	IL1RAP	LOC57228	NFAT5
AFF3	BRD4	CIT	DRAM	GAL3ST1	IL1RL1	LOC63920	NFIC
AFF4	BRMS1L	CITED2	DSCR8	GALNT10	INA	LOX	NFYB
AGPS	BRPF1	CKB	DSP	GALNT2	ING1	LRP5	NIN
AGXT	BSG	CLDN18	DVL3	GAN	INPP4A	LRRC39	NLK
AHDC1	BTBD1	CLEC3B	DYNC1I1	GATA4	INPP5A	LRRN6C	NME5
AHNAK	BUB1	CLIC2	DZIP1	GBP1	INSIG2	LTA4H	NONO
AIF1	C10orf18	CLK1	ECM2	GDF11	INSM1	LYK5	NPC1L1
AKAP13	C11orf57	CLMN	EDNRB	GENX-341	INSR	M-RIP	NRXN1
AKAP14	C14orf112	CLSTN1	EFNA1	GFPT1	INTS2	MACF1	NT5C3
ALDH4A1	C14orf147	COBLL1	EFNB2	GFRA3	IRF1	MAD2L1	NUDT15
ALDH7A1	C14orf8	COL15A1	EGFR	GLIPR1	IRF2	MAN1A1	NUMA1
ALDOA	C16orf45	CPA3	EGLN3	GMCL1	IRS1	MAP3K3	NUP43
ALDOB	C16orf72	CPA6	EIF2A	GNAI1	ISL2	MAP4	OLR1
ALKBH5	C18orf19	CPNE1	EIF2C1	GNAL	JARID1B	MAWBP	OPN3
ALOX5	C20orf19	CPNE6	EMP2	GNS	JMJD1C	MBP	OSBP2
ALPI	C5orf28	CREB1	EP400NL	GOLGA4	KBTBD11	MCAM	OSBPL10
AMDD	C5orf29	CREG2	EPB49	GPD2	KBTBD4	MDK	OSGEP
AMPD3	C6orf111	CRIM1	EPC2	GPR109B	KCMF1	MDM4	OSTF1
ANAPC5	C6orf150	CROP	ERO1L	GPR146	KCNA1	MED12	P4HA1
ANGPTL4	C9orf74	CRYAA	ET	GPSM2	KCNJ13	MEST	PAG1
ANKRD6	C9orf75	CSDE1	EYA3	GPX3	KCNN3	MFAP3L	PANK3
ANXA1	CA6	CSNK2A2	F2	GRIK1	KCTD20	MGC33894	PAPD5
ANXA4	CALCRL	CSTB	FAM13A1	GRM3	KCTD3	MKNK2	PAQR8
ANXA6	CAPN1	CTSD	FAM69B	GSTM4	KCTD9	MKX	PARS2
AP1S2	CAPN5	CUGBP2	FARP1	GTSE1	KHK	MLF1IP	PBK
AP3B2	CASKIN2	CXCL14	FATE1	H2AFX	KIAA0195	MLLT4	PCDH17
APOC3	CAST	CXCR6	FBLN1	HAMP	KIAA0828	MNAB	PCMTD1
ARF4	CBLB	CXXC5	FBLN2	HCFC1R1	KIF22	MORC2	PDCD4
ARFGEF1	CBLN2	CYLN2	FBS1	HIC2	KIF3A	MORC3	PDE1B
ARG1	CCBL1	CYP3A5	FBXO31	HLA-DQB2	KLB	MOSC2	PDGFB
ARHGEF5	CCDC45	DAZ4	FCGR2A	HLF	KNTC2	MPHOSPH1	PDK4
ARMCX4	CCL18	DBT	FETUB	HMGCS1	KPNA1	MRE11A	PEG3
ARPP19	CCNG2	DC-UbP	FGD4	HNRPH1	KPNA6	MRPS31	PELI2
ARRDC3	CCNI	DCUN1D1	FGF5	HNRPR	KYNU	MST150	PEPP-2

Table S1. List of 610 genes selected for synergy analysis (cond)

PGAP1	RBM26	SLC25A3	THNSL1	ZNF43
PHF21A	RBMX	SLC26A6	TIMP3	ZNF496
PHKA2	REEP1	SLC2A14	TLE3	ZNF532
PHLDB2	REG1A	SLC2A3	TMEM159	ZNF533
PIAS1	REV1L	SLC30A10	TMSB4X	ZNF559
PIGK	RFPL1	SLC30A3	TNXB	ZNF566
PIGS	RGS13	SLC31A1	TOMM20	ZNF650
PIPOX	RHOBTB3	SLC35C1	TOP2A	ZNF76
PITPNM1	RIPK4	SLC35E3	TOX	ZNF785
PJA2	RNASE4	SLC36A4	TPX2	ZZEF1
PKM2	RNF141	SLC39A3	TRAF3IP2	
PLCXD1	RNF19	SLC7A8	TRIM24	
PLK1	RNH1	SLFN5	TRIM6	
PLOD2	RPL18	SLIC1	TRIP10	
PLXDC1	RPL5	SLIT1	TRIT1	
PMM1	RPL6	SLPI	TSNAX	
PPFIBP1	RPS14	SMARCA1	TTYH1	
PPP1CC	RPSA	SMC3	TULP2	
PPP1R1A	RUNX1T1	SMC5	TYRP1	
PPP3CC	RUNX2	SMNDC1	UACA	
PPP6C	RYBP	SMYD2	UBE1C	
PQLC2	S100A8	SNRPD3	UBE2B	
PRDM15	SBF2	SNX25	UBE2D2	
PRKAB2	SCARB1	SOCS3	UBE2L6	
PRKCBP1	SCD5	SOCS5	UBE2U	
PRPF38B	SCEL	SORBS1	UGP2	
PRR13	SDPR	SORBS2	ULK2	
PSCD3	SEC31A	SORL1	UNQ1887	
PSD3	SEMA6A	SPAG9	UQCRC2	
PSORS1C1	SEMA6D	SPG20	USP47	
PTPRM	SERF1A	SPTA1	UTP14C	
PTPRR	SERINC2	SPTLC1	VSTM2	
PTTG1IP	SERPINA3	SSX2IP	WAC	
PURA	SERPINB1	STRBP	WDR33	
PXK	SERPINI1	STX1A	WDR45L	
PYGB	SETD3	SUV39H2	WDR5	
PYGM	SETD7	SYAP1	WDR73	
RAB27B	SETDB2	SYNJ1	WIPI2	
RAB5A	SFRS11	SYT1	WNK1	
RAB9B	SGCB	SYT11	WSB1	
RABL2B	SGEF	SYT5	WWC1	
RAD21	SGTB	TAF7L	WWC2	
RAD9B	SH3RF2	TBC1D20	XPO7	
RAF1	SIKE	TBC1D5	ZBTB10	
RASL11B	SIN3A	TBL1X	ZDHHC22	
RBBP5	SIRT4	TBX3	ZFP1	
RBJ	SKI	TCEAL4	ZHX2	
RBM10	SLC16A1	TCEAL7	ZMYM6	
RBM18	SLC1A2	TCF7L2	ZNF207	
RBM23	SLC1A7	TFAP2B	ZNF236	
