# Supplementary material for: Synergy Analysis Reveals Association between Insulin Signaling and Desmoplakin Expression in Palmitate Treated HepG2 Cells
Source: PLoS One. 2011 Nov 23;6(11):e28138. doi: 10.1371/journal.pone.0028138 (PMC3223234; doi:10.1371/journal.pone.0028138)
Supplement: Table S2 — All KEGG pathways significantly enriched in the synergy network. (DOC) [file pone.0028138.s006.doc]

Table S2. KEGG pathways enriched in synergy network

KEGG pathways Enriched in synergy network		# genes belong to the pathway		P-values of over-representations	
KEGG Entry	Pathway name	In genome	In synergy network	Before FDR adjustment	After FDR adjustment
hsa04910	Insulin signaling pathway	134	15	4.877E-09	6.682E-07
hsa04520	Adherens junction	74	10	3.057E-07	2.094E-05
hsa00310	Lysine degradation	40	7	3.162E-06	1.444E-04
hsa00330	Arginine and proline metabolism	34	6	1.549E-05	5.304E-04
hsa04916	Melanogenesis	99	8	2.028E-04	5.557E-03
hsa04120	Ubiquitin mediated proteolysis	131	9	2.820E-04	6.439E-03
hsa04010	MAPK signaling pathway	260	13	3.362E-04	6.580E-03
hsa05210	Colorectal cancer	84	7	4.180E-04	7.158E-03
hsa04310	Wnt signaling pathway	148	9	6.879E-04	9.424E-03
hsa04810	Regulation of actin cytoskeleton	210	11	6.443E-04	9.807E-03
hsa04360	Axon guidance	127	8	1.075E-03	1.339E-02
hsa00010	Glycolysis / Gluconeogenesis	62	5	3.225E-03	3.156E-02
hsa00380	Tryptophan metabolism	60	5	2.794E-03	3.190E-02
hsa05215	Prostate cancer	89	6	3.182E-03	3.353E-02
hsa00051	Fructose and mannose metabolism	41	4	4.193E-03	3.590E-02
hsa04660	T cell receptor signaling pathway	93	6	3.956E-03	3.613E-02
hsa00620	Pyruvate metabolism	42	4	4.576E-03	3.688E-02
hsa05218	Melanoma	70	5	5.433E-03	3.722E-02
hsa00710	Carbon fixation	23	3	5.776E-03	3.768E-02
hsa04930	Type II diabetes mellitus	44	4	5.412E-03	3.902E-02
hsa00280	Valine, leucine and isoleucine degradation	44	4	5.412E-03	3.902E-02
hsa04110	Cell cycle	112	6	9.637E-03	6.001E-02
hsa00220	Urea cycle and metabolism of amino groups	30	3	1.219E-02	6.957E-02
hsa05217	Basal cell carcinoma	55	4	1.184E-02	7.055E-02
hsa04012	ErbB signaling pathway	87	5	1.330E-02	7.290E-02
hsa00640	Propanoate metabolism	33	3	1.581E-02	8.330E-02
hsa04540	Gap junction	95	5	1.881E-02	9.205E-02
hsa04662	B cell receptor signaling pathway	63	4	1.872E-02	9.501E-02
hsa04720	Long-term potentiation	67	4	2.293E-02	1.083E-01
hsa04115	p53 signaling pathway	68	4	2.406E-02	1.099E-01
hsa05211	Renal cell carcinoma	69	4	2.522E-02	1.115E-01
hsa00770	Pantothenate and CoA biosynthesis	16	2	2.672E-02	1.144E-01
hsa04630	Jak-STAT signaling pathway	153	6	3.785E-02	1.482E-01
hsa04510	Focal adhesion	195	7	3.915E-02	1.490E-01
hsa05020	Parkinson's disease	20	2	4.057E-02	1.502E-01
hsa00071	Fatty acid metabolism	46	3	3.777E-02	1.522E-01
hsa01030	Glycan structures - biosynthesis 1	118	5	4.232E-02	1.526E-01
hsa05030	Amyotrophic lateral sclerosis (ALS)	19	2	3.690E-02	1.532E-01
hsa00563	Glycosylphosphatidylinositol(GPI)-anchor biosynthesis	21	2	4.437E-02	1.559E-01
hsa00785	Lipoic acid metabolism	3	1	4.751E-02	1.587E-01
hsa00562	Inositol phosphate metabolism	51	3	4.889E-02	1.595E-01
hsa04210	Apoptosis	84	4	4.679E-02	1.603E-01

* FDR: false discovery rate
