# Supplementary material for: Synergy Analysis Reveals Association between Insulin Signaling and Desmoplakin Expression in Palmitate Treated HepG2 Cells
Source: PLoS One. 2011 Nov 23;6(11):e28138. doi: 10.1371/journal.pone.0028138 (PMC3223234; doi:10.1371/journal.pone.0028138)
Supplement: Table S3 — All neighbor genes significantly associated with insulin signaling. (DOC) [file pone.0028138.s007.doc]

Table S3. Neighbor genes of Insulin signaling pathways in synergy network

Neighbor genes	Degree in synergy network	# insulin signaling genes connected	Neighbor genes	Degree in synergy network	# insulin signaling genes connected
HOMER2	180	8	DPYD	18	2
LRRC39	132	6	SBF2	18	2
ACSS2	174	6	MLLT4	19	2
NPC1L1	97	5	SYNJ1	19	2
ACADSB	12	3	ANKRD6	20	2
DSP	12	3	GAN	20	2
PANK3	15	3	LTA4H	20	2
SLC39A3	18	3	PTPRM	20	2
ICA1	20	3	BUB1	21	2
TTYH1	30	3	KIAA0195	21	2
EP400NL	31	3	TULP2	21	2
EGFR	36	3	CRIM1	22	2
C6orf150	37	3	BBX	23	2
FAM69B	37	3	SLC2A14	23	2
PDCD4	47	3	SPAG9	23	2
76P	52	3	ALDOA	25	2
ET	58	3	OSBP2	26	2
ZNF566	64	3	SMYD2	26	2
ANGPTL4	65	3	ZMYM6	26	2
C9orf74	89	3	TOMM20	27	2
NAG	160	3	BRD4	28	2
LOC283588	3	2	DDIT4	28	2
KNTC2	4	2	FUT11	28	2
ALDH4A1	5	2	PEG3	28	2
DIDO1	6	2	PIPOX	33	2
SNX25	6	2	MXI1	34	2
CDC14B	7	2	LOX	35	2
CKB	7	2	RAD21	35	2
GMCL1	7	2	C6orf111	40	2
PLK1	7	2	ZHX2	41	2
UBE2L6	7	2	SH3RF2	44	2
BMP4	9	2	RNF19	46	2
C18orf19	9	2	MST150	49	2
HNRPR	9	2	EFNA1	50	2
COBLL1	10	2	RBM18	52	2
HPN	10	2	HNRPU	53	2
MOSC2	10	2	ANXA4	55	2
AHDC1	11	2	AP1S2	70	2
BAZ2B	11	2	C16orf45	72	2
CCBL1	11	2	MACF1	73	2
FARP1	11	2	INSIG2	98	2
BIRC5	12	2	BRPF1	1	1
DKFZp762E131[illegible]	12	2	JARID1B	1	1
HRASLS3	12	2	KHK	1	1
STRBP	12	2	S100A8	1	1
DC-UbP	13	2	AHNAK	2	1
RNASE4	13	2	CTSD	2	1
SLC25A3	14	2	GAB1	2	1
ATAD2	16	2	ANXA1	3	1
ATXN3	16	2	APOC3	3	1
AUH	16	2	IER3	3	1
EPC2	16	2	SEMA6A	3	1
CSTB	17	2	TOP2A	3	1
GOLGA4	17	2	ARG1	4	1
NEK4	17	2	CRYAA	4	1
SCARB1	17	2	DZIP1	4	1
UGP2	17	2	UBE2D2	4	1

Table S3. Neighbor genes of Insulin signaling pathways in synergy network (cond)

Neighbor genes	Degree in synergy network	# insulin signaling genes connected	Neighbor genes	Degree in synergy network	# insulin signaling genes connected
ABHD6	5	1	FLJ10154	13	1
C14orf147	5	1	IRF1	13	1
CLMN	5	1	MTDH	13	1
LARP4	5	1	MTTP	13	1
RASL11B	5	1	SLC26A6	13	1
WDR33	5	1	UACA	13	1
ALKBH5	6	1	UQCRC2	13	1
CASKIN2	6	1	BSG	14	1
CDCA1	6	1	FGFR1	14	1
CSNK2A2	6	1	MTMR10	14	1
ERO1L	6	1	P4HA1	14	1
FBLN1	6	1	CA6	15	1
NONO	6	1	CYLN2	15	1
PJA2	6	1	FAM13A1	15	1
PMM1	6	1	GFRA3	15	1
SSX2IP	6	1	PCMTD1	15	1
WSB1	6	1	PQLC2	15	1
ABHD5	7	1	PRR13	15	1
CCNG2	7	1	SERINC2	15	1
DHRS8	7	1	SERPINB1	15	1
PAPD5	7	1	SORBS2	15	1
PTTG1IP	7	1	AKAP14	16	1
RBM10	7	1	AMPD3	16	1
SMARCA1	7	1	DNAJC4	16	1
ACBD4	8	1	DRAM	16	1
ALDH7A1	8	1	FGF5	16	1
AURKA	8	1	RPS14	16	1
C20orf19	8	1	ADM	17	1
MAD2L1	8	1	ARRDC3	17	1
MTSS1	8	1	CCDC45	17	1
PBK	8	1	CDC2L5	17	1
C14orf112	9	1	ING1	17	1
PIGS	9	1	RABL2B	17	1
TRIM24	9	1	SCEL	17	1
AADAT	10	1	ALPI	18	1
NT5C3	10	1	KPNA6	18	1
RBM26	10	1	NFAT5	18	1
SERPINI1	10	1	PDGFB	18	1
SMNDC1	10	1	VSTM2	18	1
TBC1D20	10	1	CPNE1	19	1
C9orf75	11	1	FBLN2	19	1
DBT	11	1	GRIK1	19	1
MAWBP	11	1	PELI2	19	1
PRKCBP1	11	1	RIPK4	19	1
RPL18	11	1	SLC2A3	19	1
EYA3	12	1	TCF7L2	19	1
KIF22	12	1	AFF3	21	1
LMAN1L	12	1	CCNI	21	1
LRP5	12	1	ALDOB	22	1
MEST	12	1	GPR146	22	1
MNAB	12	1	CROP	23	1
NUMA1	12	1	CXXC5	23	1
RAB9B	12	1	FN1	23	1
SGEF	12	1	LIAS	24	1
SMC5	12	1	PLOD2	24	1
TLE3	12	1	KCTD3	25	1

Table S3. Neighbor genes of Insulin signaling pathways in synergy network (cond)

Neighbor genes	Degree in synergy network	# insulin signaling genes connected
MGC33894	25	1
USP47	25	1
CAPN1	26	1
EGLN3	26	1
PIGK	26	1
PRPF38B	26	1
MAP4	27	1
SLC1A7	27	1
EPB49	28	1
GPD2	29	1
NIN	29	1
UTP14C	29	1
BNIP3L	30	1
C14orf8	30	1
PAQR8	30	1
SLC35E3	30	1
HIC2	31	1
WWC1	32	1
IFITM1	33	1
DYNC1I1	36	1
HCFC1R1	36	1
KPNA1	36	1
CITED2	38	1
ZNF207	42	1
ISL2	43	1
IGFBP7	48	1
NLK	48	1
PPP6C	49	1
RAB5A	50	1
TSNAX	50	1
OSTF1	53	1
WDR5	55	1
MKX	60	1
DDX17	74	1
BTBD1	77	1
KYNU	80	1
